# Feasibility and Safety of Bariatric Surgery in High-Risk Patients: A Single-Center Experience

**DOI:** 10.1155/2018/7498258

**Published:** 2018-01-14

**Authors:** Yusef Moulla, Orestis Lyros, Matthias Blüher, Philipp Simon, Arne Dietrich

**Affiliations:** ^1^Department of Visceral, Transplant, Thoracic and Vascular Surgery, University of Leipzig, Liebigstraße 20, 04103 Leipzig, Germany; ^2^Integrated Treatment and Research Centre (IFB) for Obesity Diseases, Philipp-Rosenthal-St. 27, 04103 Leipzig, Germany; ^3^Department of Internal Medicine, University of Leipzig, Liebigstraße 20, 04103 Leipzig, Germany; ^4^Department of Anesthesia and Intensive Care Medicine, University of Leipzig, Liebigstraße 20, 04103 Leipzig, Germany

## Abstract

**Introduction:**

Despite the feasibility and safety of bariatric procedures nowadays, high-risk patients with vast obesity and severe comorbidities demonstrate relatively high perioperative morbidity and mortality rates and, therefore, form a distinguished challenge for the bariatric surgeons.

**Methods:**

We retrospectively analyzed high-risk patients, who underwent bariatric surgery in University Hospital Leipzig between May 2012 and December 2016. High-risk patients were defined when (Bergeat et al., 2016) at least one of the following risk factors was met: age ≥ 70 years, body mass index (BMI) > 70 kg/m^2^, liver cirrhosis, end-organ failure, or immunosuppression by status after organ transplantation along with (Birkmeyer et al., 2010) at least two comorbidities associated with obesity. Our analysis included early postoperative complications.

**Results:**

A total of 25 high-risk obese patients were identified. All patients had a standardized postoperative management with a mean length of hospital stay of 4 ± 1.4 days. One patient required an operative revision due to a stapler line leak after sleeve gastrectomy. No other major postoperative complications occurred.

**Conclusion:**

Bariatric surgery for severe high-risk patients can be performed safely in high-volume centers following standardized procedures.

## 1. Introduction

Bariatric surgery is the most effective therapy for morbid obesity in order to achieve sustained weight loss [1]. Additionally, it is reported that bariatric surgery significantly improves the quality of life and leads in complete remission or at least in partial improvement of comorbidities associated with obesity [2]. In high-risk obese patients with severe health conditions or comorbidities, bariatric surgery appears to have a positive impact; however, it is associated with increased perioperative morbidity and mortality rates and therefore consists of a distinguished challenge for the bariatric surgeon [1–5].

Laparoscopic Roux-en-Y gastric bypass (LRYGB) and laparoscopic sleeve gastrectomy (LSG) are internationally recognized as safe and feasible bariatric operations [3, 6]. According to data from the IFSO-European Chapter Centre, postoperative complication rates are 3.02% and 2.12% after LRYGB and LSG, respectively. Mortality following bariatric surgery is usually within a range of 0 to 1.5% in typical obese patients [7–9]. However, bariatric surgery in high-risk patients has potentially an increased morbidity and mortality when compared with obese patients not of increased risk [9, 10]. According to these data, the mortality in obese patients with high risk is 17-fold greater compared to no-risk patients [9].

Various reports have already attempted to describe the profile of high-risk patients in bariatric surgery and evaluate its safety. Mognol et al. and Cottam et al. described the patients with BMI > 60 kg/m^2^ and major co-morbidities such as diabetes mellitus (DM) type 2, asthma, or obstructive sleep apnea (OSA) as high-risk patients und reported that LSG can be safely performed in those patients [11, 12]. However, the factors that form a high-risk profile for bariatric patients are not largely defined yet and in the existing literature vary a lot. Also, BMI rates of 60 to 70 kg/m^2^ are routine for many surgeons.

## 2. Patients and Methods

### 2.1. Study Population

We retrospectively analyzed high-risk patients undergoing bariatric surgery in our center for bariatric and metabolic surgery. Between May 2012 and December 2016, 450 patients underwent bariatric surgery; among those were 25, who matched with the following criteria for high risk. High-risk patients were defined those who had one of the risk factors summarized in Table 1 and at least two of obesity-associated comorbidities such as DM Type 2, arterial hypertension, OSA, or chronic obstructive pulmonary disease (COPD)/asthma. All patients who fulfilled these criteria were included. The diagnosis of liver cirrhosis was confirmed histologically through a simultaneous laparoscopic biopsy intraoperatively. The function of the liver in cirrhotic patients was assessed by the Child–Pugh classification.

### 2.2. Surgical Procedures

Two types of bariatric operations were performed, either LSG or LRYGB. Patients did not receive any premedication at the day of surgery. Anesthesia was induced using propofol and infusion of remifentanil. Rocuronium was given to facilitate tracheal intubation. Standardized balanced anesthesia was maintained with volatile desflurane and continuous infusion of remifentanil. The mechanical ventilation was performed according to the German anesthesiologic standards. All patients were given after the introduction of the anesthesia a single-shot antibiotic according to the German guidelines of perioperative antibiotic prophylaxis. Thromboembolic prophylaxis was performed intraoperatively using pneumatic compression and postoperatively through a low-molecular-weight heparin at a dose adapted to the patient's body weight, which was started 6 hours after the operation. Additional prophylaxis was provided postoperatively by using gradual compression stockings and early full mobilization, starting at the evening of the operation day. Proton pump inhibitors were started the day after the surgery and maintained for at least 6 weeks. All operations were performed laparoscopically by the same bariatric surgeon.

For *LSG*, the lesser sac was entered by dividing the gastroepiploic vessels along the greater curvature. The stomach was transacted using a stapler along a 34F bogie. The transection was started 4-5 cm prior the pylorus along the greater curvature towards the ankle of HIS.

For *LRYGB*, a 20–30 ml gastric pouch was created using a stapler. The alimentary limb (antecolic) was 150–170 cm, and the biliopancreatic limb was 50–80 cm long, depending on the BMI. The anastomosis (gastrojejunostomy) as well as the jejunojejunostomy was performed with a linear stapler and suturing of the defect.

### 2.3. Postoperative Management and Follow-Up

All patients, except the patient with terminal renal failure, received postoperatively the same standard infusion therapy, 3 liters of crystalloid fluid (Ringer's solution) at the first postoperative day, and 2 liters at the second and third postoperative days. All patients began to drink water at the first postoperative day and were allowed to take clear soup at the third postoperative day. The drain was removed before discharge at day 4 postoperatively. Following discharge, all patients received soft nutrition for at least 3 weeks, and small meals were recommended.

The morbidity and mortality rates were analyzed for a period of 30 days postoperatively. All patients had an outpatient visit after 12 and 48 days postoperatively. The severity of the postoperative complications was categorized according to the Clavien–Dindo classification [13, 14].

### 2.4. Statistical Analysis

Categorical data are expressed as absolute or relative frequencies. Continuous data are expressed as median and interquartile range or mean and standard deviation. The statistical descriptive analysis was performed through SPSS Version 20.0.

## 3. Results

**Figure 1:**
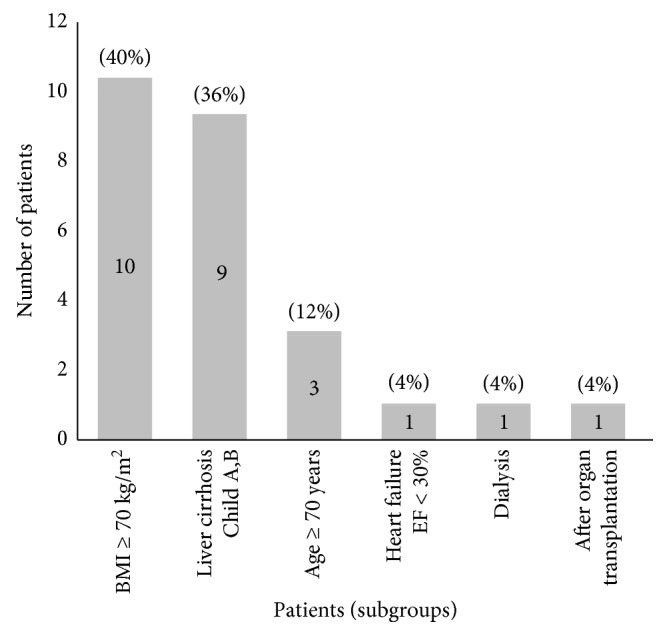
The number of patients in each high-risk subgroup (%).

Between May 2012 and December 2016, a total of 25 high-risk patients undergoing bariatric surgery were identified (Figure 1).

Among the 25 patients, there were 11 (44%) females and 14 (56%) males. The mean age was 50.9 ± 13.8 years. LSG was performed in 14 patients (56%) and LRYGB in 11 patients (44%). The median duration of the surgery was 152 min (range 79 to 310 min) and was analogous to the no-risk group (median 160 min). The characteristics of the study cohort and the incidence of additional comorbidities such as diabetes mellitus (DM), hypertension, gastroesophageal reflux disease, OSA, and COPD are summarized in Table 2.

### 3.1. Postoperative Complications

The length of stay at hospital was in mean 4.4 ± 1.4 days. Twenty-three patients (92%) could be discharged at the fourth day postoperatively, as scheduled via standards. One patient was discharged two days later due to unclear high inflammatory laboratory values. The vital signs were stable. The control laboratory results returned to the normal values, and the patient discharged at the 6th postoperative day without complications. One patient had a stapler line leak two days after LSG and underwent revision by relaparoscopy on the same day. The leak was closed with a running suture. This patient was discharged without any further problems on the 10th postoperative day. Four patients including this patient with stapler leak had a postoperative mild wound infection in one trocar position. Those wound infections were treated without problems or any intervention in the outpatient's department postoperatively. One patient suffered a temporary postoperative lesion of the right fibular nerve through compression socks. This patient was discharged standardized at the 4th postoperative day, and this lesion was completely regressive 6 weeks later.

**Figure 2:**
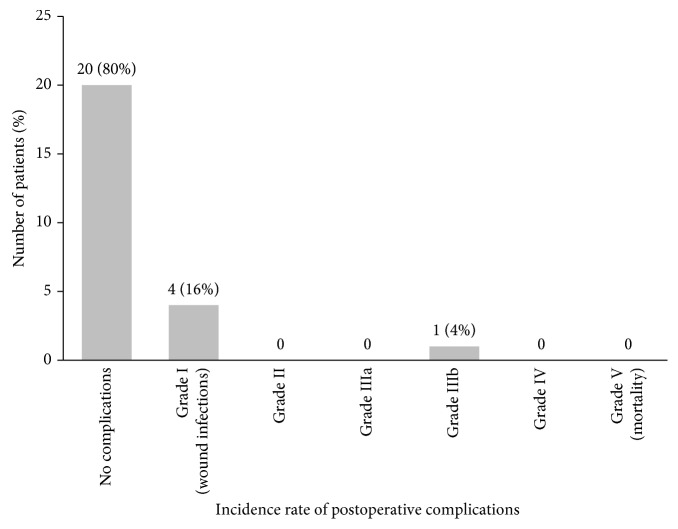
The rate of postoperative complications within 30 days after the surgery in the high-risk group.

The rate of the major complications, Grade IIIb, was 4%. The distribution of the postoperative complications within the first postoperative month according to the Clavien–Dindo classification [14] is demonstrated in Figure 2.

No patient demonstrated postoperative bleeding, acute renal failure, pneumonia, vein thrombosis, respiratory failure, acute liver failure, and pulmonary thrombosis within the first 30 days following the operation, and the mortality rate was 0%.

### 3.2. Subgroup Analysis

#### 3.2.1. BMI

In the subgroup of patients with BMI ≥ 70 kg/m^2^, LSG was performed in the majority of the patients (90%, *n* = 9/10). This procedure was performed as a first step within a two-stage concept as described by Regan et al. in 2003 [15]. In three patients of this subgroup, a previous endoscopic therapy (1x EndoBarrier™ Therapy, 2x Gastric Balloon) had failed. One patient with BMI ≥ 70 kg/m^2^ underwent a LRYGB without postoperative complications and was discharged on the 4th postoperative day as planned. For BMI ≥ 70 kg/m^2^ patients undergoing LSG, their mean weight was 217 kg (range 189–249 kg) and the mean operative time was 137 min (range 79–310 min).

#### 3.2.2. Liver Cirrhosis

Among all patients who underwent bariatric surgery between May 2012 and December 2016, we found 9 patients (2%) with liver cirrhosis. Liver cirrhosis was unknown before surgery in 6 patients (1.3%) and was confirmed through a simultaneous biopsy during the operation. One patient with known liver cirrhosis preoperatively suffered from Child B cirrhosis and the other two from Child A. LRYGB was performed in 6 patients and LSG in 3 patients out of the cirrhosis subgroup. No bleeding or liver decompensation was observed postoperatively.

## 4. Discussion

There are a continuously increasing number of obese patients with an indication for bariatric surgery. A certain number of those demonstrate severe health conditions or comorbidities, which categorize them as high-risk patients for bariatric surgery. The definition of risk factors in bariatric surgery is very variable. DeMaria et al. [16, 17] suggested a mortality risk score (OS MRS) to define the high-risk patients in bariatric surgery. The risk factors according to this report were age > 45 years, BMI ≥ 50 kg/m^2^, arterial hypertension, and male gender along with risk factors for pulmonary thromboembolism. According to this scale, high-risk patients were defined as those patients who demonstrated four or five of the predefined risk factors [16].

Campos et al. [18] reported that diabetes mellitus, early surgeon experience, and open surgery were also detected in particular as significant risk factors for early postoperative complications. Furthermore, several reports have attempted to form score systems in order to predict the perioperative risk in bariatric patients [19, 20]. In a systemic review by Buchwald et al. [21], BMI ≥ 50 kg/m^2^, age > 65 years, and the male gender were considered as risk factors for early postoperative morbidity following bariatric surgery. Despite the higher early morbidity rates following bariatric surgery in high-risk patients as defined in those reports, bariatric surgery could be performed with accepted safety. In our study, we included patients demonstrating more severe risk factors such as liver cirrhosis or advanced heart failure (EF < 30%), which can further complicate the perioperative management and increase the morbidity and mortality rates in bariatric surgery.

Although in patients who are super-super obese, LSG is the procedure of choice, LRYGB remains the gold standard in bariatric surgery as it offers a good compromise between long-term effectiveness and safety [6, 13, 22]. The rate of early postoperative complications for LRYGB is higher than that for LSG [3]. In high-risk patients undergoing LRYGB, various reports have shown increased rates of early postoperative complications compared to no-risk patients, depending on the institution's surgical experience and how the risk factors were defined. However, the early morbidity (<30 days) and the major early complication rates following LRYGB were found in all studies acceptably low (ca. 3%) [3, 13]. Similarly, in our current study, we have not found early major postoperative complications following LRYGB.

The effectiveness of the LSG as a first procedure was described in many studies [3, 11, 12]. In our study, most of the patients with BMI ≥ 70 kg/m^2^ underwent LSG (90%) mainly as the first step in a two-stage concept approach as described by Regan et al. [15]. Mognol et al. [12] published similar data and described LSG as a safe procedure for patients with BMI ≥ 60 kg/m^2^. Despite the small size of patients in the present study, our results show similarly low early major complication rates in the patients who are severely obese undergoing LSG as described in previous reports [11, 15, 23]. Although the patient's number with BMI ≥ 70 kg/m^2^ is low in our cohort, LSG appears as a safe primary procedure for handling patients with extremely high BMI. We had one stapler line leak, which is a known complication of LSG, occurring in 1–3%.

Both the procedures LSG and LRYGB were already described as well-tolerated operations in patients with compensated liver cirrhosis [22]. Obesity is strongly associated with nonalcoholic liver disease (NASH) 25–55% [24]. Up to 25% of patients with NASH can progress into liver cirrhosis [22, 25]. Bariatric surgeons often face unexpected liver cirrhosis during the operation in up to 2% [26], in our series in 1.3%. In such cases, it is of high importance to consider the patients' management and the intraoperative evaluation of whether to continue and perform the operation or not. In our opinion, bariatric surgery can be performed in the absence of signs of Child C liver cirrhosis and signs of severe portal hypertension such as ascites and large vein collateralization. LSG still remains an acceptable option in such patients and can be performed safely as also reported by Shimizu et al. [22]. Despite the limited number of patients, our study confirms that even LRYGB can be done safely in selected cases.

Our data suggest that standardized bariatric surgery in experienced high-volume centers can be performed safely even in high-risk patients with severe comorbidities. Our defined risk factors in this study are more severe compared with previous reports, which deal with the issue of high-risk factors in bariatric patients. By following standardized procedures through an expert surgical team, bariatric surgery can be safely performed even in patients with severe risk factors with low early morbidity and mortality rates as described by Birkmeyer et al. [27]. Regarding the procedure, LRYGB can be done safely in the patients with liver cirrhosis or other severe comorbidities in selected cases, while LSG is a safe option in patients who are severely obese (BMI ≥ 70 kg/m^2^).

Some limitations should be considered in this study: first, the descriptive nature of this study; second, the small number of the patients; and lastly, the absence of the control group of no-risk patients to compare with that of our high-risk patients.

## 5. Conclusion

Our data suggest that standardized bariatric surgery in high-volume centers can be performed safely in advanced high-risk patients. Regarding the procedure, LRYGB can be done safely in the patients with liver cirrhosis or other severe comorbidities in selected cases, while LSG is a safe option in patients who are super-super obese.

## Figures and Tables

**Table 1:** Definition of high risks.

Age	≥70 years
BMI	≥70 kg/m^2^
Heart failure	EF < 30%
Liver cirrhosis	Child A, B
End-stage renal failure	Dialysis (+)
Organ transplantation	After organ transplantation

BMI = body mass index; EF = ejection fraction.

**Table 2:** Baseline of study cohort.

Patient characteristics	Cohort (*N* = 25)
Age, years	50.9 ± 13.8
Sex (male), *n* (%)	14 (56%)
Operation type	
(i) LRYGB	11 (44%)
(ii) LSG	14 (56%)
Duration of surgery, min	152 (range 79–310)
BMI, kg/m^2^	59 (range 38–87)
ASA classification	3 (range 2–4)
Metabolic comorbidities	
Hypertension	24 (96%)
Diabetes mellitus type 2	18 (72%)
Arthrosis	13 (52%)
Obstructive sleep apnea (OSA)	9 (36%)
Asthma/COPD	7 (28%)
GERD	4 (16%)
Previous DVT	1 (4%)

Entries are medians (range) or numbers (%); LRYGB = laparoscopic Roux-en-Y gastric bypass; LSG = laparoscopic sleeve gastrectomy; BMI = body mass index; GERD = gastroesophageal reflux disease; DVT = deep vein thrombosis.
